# X-ray repair cross-complementing protein 1 (XRCC1) loss promotes β-lapachone –induced apoptosis in pancreatic cancer cells

**DOI:** 10.1186/s12885-021-08979-y

**Published:** 2021-11-17

**Authors:** Yansong Zheng, Hengce Zhang, Yueting Guo, Yuan Chen, Hanglong Chen, Yingchun Liu

**Affiliations:** 1 Department of Hepatobiliary Surgery, First Affiliated Hospital of Fujian Medical University, 20 Chazhong Road, Fuzhou, 350005 China; 2 Key Laboratory of Stem Cell Engineering and Regenerative Medicine, Fujian Province University/School of Basic Medical Science, Fujian Medical University, Fuzhou City, 350122 Fujian Province China; 3 Fujian University of Traditional Chinese Medicine, Fuzhou City, 350122 Fujian Province China

**Keywords:** Pancreatic cancer, XRCC1, DNA BER, Apoptosis

## Abstract

**Background:**

β-lapachone (β-lap), the NQO1 bioactivatable drug, is thought to be a promising anticancer agent. However, the toxic side effects of β-lap limit the drug use, highlighting the need for a thorough understanding of β-lap’s mechanism of action. β-lap undergoes NQO1-dependent futile redox cycling, generating massive ROS and oxidative DNA lesions, leading to cell death. Thus, base excision repair (BER) pathway is an important resistance factor. XRCC1, a scaffolding component, plays a critical role in BER.

**Methods:**

We knocked down XRCC1 expression by using pLVX-shXRCC1 in the MiaPaCa2 cells and BxPC3 cells and evaluated β-lap-induced DNA lesions by γH2AX foci formation and alkaline comet assay. The cell death induced by *XRCC1* knockdown + β-lap treatment was analysed by relative survival, flow cytometry and Western blotting analysis.

**Results:**

We found that knockdown of *XRCC1* significantly increased β-lap-induced DNA double-strand breaks, comet tail lengths and cell death in PDA cells. Furthermore, we observed combining *XRCC1* knockdown with β-lap treatment switched programmed necrosis with β-lap monotherapy to caspase-dependent apoptosis.

**Conclusions:**

These results indicate that XRCC1 is involved in the repair of β-lap-induced DNA damage, and XRCC1 loss amplifies sensitivity to β-lap, suggesting targeting key components in BER pathways may have the potential to expand use and efficacy of β-lap for gene-based therapy.

**Supplementary Information:**

The online version contains supplementary material available at 10.1186/s12885-021-08979-y.

## Background

Pancreatic cancer is classified as pancreatic ductal adenocarcinoma (PDA) due to more than 90% of pancreatic cancer having ductal morphology [1]. PDA is currently ranked as the fourth leading cause of cancer mortality in the United States [2]. The main cure for PDA is surgical resection with low survival rates [3]. Therefore, it is imperative to develop novel effective strategies to treat PDA. Current chemotherapy achieves significant clinical benefit, but lacking tumor-selectivity often results in undesirable off-target toxicities in normal, healthy tissue, highlighting the need for gene-based treatment [4].

β-lapachone (β-lap, in clinical form, ARQ761), a tumor-selective drug, is a NAD(P)H: quinone oxidoreductase 1 (NQO1) bioactivatable drug. NQO1 is highly expressed in many cancers. 90% of pancreatic and NSCLC cancers have 10- to 100-fold of elevated levels of NQO1, and 60% of breast, prostate and colon cancers overexpress NQO1 5- to 20- fold [5–7]. Therefore, β-lap is a promising drug for selective treatment of not only pancreatic cancers, but many other NQO1 high tumors. β-lap’s therapeutic efficacy mainly stems from β-lap-induced futile redox cycling in NQO1^+^ cancer cells, producing oxidative DNA lesions and consequent cell death, while normal tissues are spared by low NQO1 expression [7, 8]. Currently β-lap has been used in a clinical trial, but there are still some problems that limit the drug use. Huang et al. reported that exposure to high dose of β-lap caused severe muscle contractions, labored breathing, and lethality in some cases of A549-bearing mice [7]. Therefore, strategies to enhance its efficacy without augmenting toxicity are needed.

Base excision repair (BER) pathway plays pivotal roles in fixing DNA base lesions and single strand breaks (SSBs). We speculated blocking BER can enhance response to β-lap. X-ray repair cross-complementing protein 1 (XRCC1), a scaffolding component, plays a critical role in BER. XRCC1 interacts and forms a complex with DNA ligase III, polymerase beta and poly (ADP-ribose) polymerase during base excision repair and SSB repair [9]. XRCC1 loss is reported to be extremely hypersensitive to multiple oxidative agents due to repair deficiency [9, 10].

We, therefore, hypothesized that XRCC1 lack would significantly increase β-lap-induced DNA damage in a tumor-selective manner that in turn decrease β-lap uptake. To test our hypothesis, we examined the reactive oxygen species (ROS) level, DNA damage and cell death in β-lap-treated XRCC1-deficient MiaPaCa2 cells. We further investigated the underlying molecular mechanism.

## Methods

### Cell culture and drug treatment

MiaPaCa2, BxPC3, HS766T, S2–013 and PANC1 cells were obtained from the Cell Bank of Type Culture Collection (Chinese Academy of Sciences, Shanghai, China). MiaPaCa2 *NQO1*^*−*^, S2–013 *NQO1*^*+*^ and PANC1 *NQO1*^*+*^ stable cell lines were kindly gifted by Department of Biochemistry and Molecular Biology, Indiana University (Indianapolis, IN, USA). Cells were incubated in Dulbecco’s Modified Eagle’s Medium (DMEM; Hyclone; Logan, UT, USA) supplemented with 10% fetal bovine serum (FBS; Hyclone; Logan, UT, USA) at 37 °C. β-lap stocks in DMSO were stored at − 80 °C. For XRCC1 knockdown, plasmid constructs for stable depletion of human *XRCC1* mRNA were obtained from Addgene (pLVX-shRNA2), and shRNA against XRCC1 were used: CCGGCGATACGTCACAGCCTTCAATCTCGAGATTGAAGGCTGTGACGTATCGTTTTT. cells were transfected with control or pLVX-shXRCC1. XRCC1 knockdown was confirmed by Western blotting.

### Chemicals and reagents

β-lap was gifted by Department of Biochemistry and Molecular Biology, Indiana University (Indianapolis, IN, USA) and stock solutions were prepared at 50 mM in DMSO. Dicoumarol and hoechst 33258 were purchased from Sigma-Aldrich. zVAD-fmk were obtained from Merck millipore (Bedford, MA, USA).

### Western blotting

Total protein was extracted from cells and quantified. The same amount of total proteins was resolved in 4 to 20% Tris–Glycine gel, and the subsequent immunoblot was performed as previously described [3]. Antibodies used for Western blotting, included: NQO1 (1:1000; #3187), cleaved caspase 7 (1:1000; #8438), cleaved caspase 3 (1:1000; #9664), p-cdc2 (Tyr15)(1:1000; #9111), p-cdc25c (Ser216)(1:1000; #4901), p-Chk2 (Thr68)(1:1000; #2197), Chk2 (1:2000; #6334), cdc25c (1:1000; #4688), and XRCC1 (1:1000; #2735) from Cell Signaling Technology (Danvers, MA, USA), p53 (1:1000; SC-126, Santa Cruz, La Jolla, CA), γH2AX (Ser139, 1:2000; JBW301, Millipore, Temecula, CA), PARP1 (1:1000; SC-8007, Santa Cruz, La Jolla, CA), PAR (11,000; Trevigen, Gaithersburg, MD), while rabbit anti-β-actin (13,000; #4970, CST, Danvers, MA, USA) and mouse anti-α-Tubulin (15,000; #3873, CST, Danvers, MA, USA) antibodies served as loading controls.

### Immunocytochemical staining

Immunocytochemical staining of cells was performed as previously reported [11]. Briefly, cells were seeded onto 24-well plates at a density of 1.6 × 10^5^ cells per well, rinsed with PBS and fixed in methanol for 30 min.

Cells were permeabilized with 0.5% Triton X-100 for 15 min. Fixed cells were blocked with 5% goat serum in PBS for 1 h at room temperature and incubated in rabbit anti- γH2AX (Ser139) (1:500) or mouse anti-rabbit XRCC1(1:200) at 4 °C overnight. Cells were then incubated in DyLight594-conjugated (1:200; EarthOx; San Francisco, CA, USA) and DyLight488-conjugated antibodies (1:200; EarthOx; San Francisco, CA, USA) at room temperature for 1 h, and counterstained with DAPI (Invitrogen; Carlsbad, CA, USA) to visualize nucleus.

### DNA survival assays

Cells were seeded at 1× 10^4^ cells/well in a 48-well plate and allowed to attach overnight. Cells were then treated for 2 h with various β-lap doses (0–7 μmol/L), with or without dicoumarol (50 μmol/L) in 6 replicates/dose. After 2 h, the drug was removed and replaced with 400 μL of 5% FBS DMEM. After 7 days (or until 90% confluence for the untreated control), the medium was removed, and cells were washed with PBS. The PBS was discarded, and 140 μL of distilled H_2_O was added to each well. Cells were lysed by the freeze-thaw method and stained with 280 μL of Hoechst dye (from a stock of 10 μL of Hoechst 33258 (Invitrogen by thermos Fisher Scientific) in 10 mL of TNE buffer), and cells were incubated in the dark for 2 h at room temperature. DNA content was quantified by fluorescence (460 nm) in a Victor X3 plate reader (PerkinElmer Life Sciences).

### ATP / H_2_O_2_/ NAD^+^ assessments

Changes in ATP, hydrogen peroxide (H_2_O_2_), and NAD^+^ levels were assayed after drug treatments (2 h) in the absence or presence of 50 μM dicoumarol by using CellTiter-Glo, ROS-Glo and NAD/NADH-Glo assays (Promega, Madison, WI).

### Comet assay

The experiments were conducted following the manufacturer’s instructions (Alkaline comet assay kit, Trevigen; Gaithersburg, MD, USA). Briefly, after 2 h incubation with 3 μM β-lap, 10 μL of the MiaPaCa2 shScr and shXRCC1 cells (500cells/ μL) were embedded into 100 μL low melting agrose, fixed onto comet assay slides. Then slides were immersed in lysis buffer at 4 °C for 45 min, followed in pre-chilled alkaline buffer (1 mM EDTA, 300 mM NaOH, pH > 13) and subjected to alkaline electrophoresis (30 min, 1 V/cm). After staining with Propidium Iodide (PI), the images were captured by fluorescence microscopy, and results were analyzed by CASP software. The comet tails from 50 cells were measured and analyzed.

### Flow cytometry

Cells were treated with 3 μM or 6 μM β-lap for 2 h. Drug-containing media was removed and cells were incubated in fresh complete media for indicated time. For apoptosis analyses, cells were collected and stained by PI and FITC conjugated Annexin (TACS Annexin V-FITC kit, R&D system; Minneapolis, Minnesota, United States), performed according to the suggestions of the manufacturer. For cell cycle assay, after fixing cells in 100% methanol, samples were washed and re-suspended in PBS buffer containing 100 μg/mL PI, 1% Triton X-100 and 10 μg/mL RNase. Then, Cells were analyzed on a FACSAria (BD Biosciences, San Jose, CA) and cell distributions were modeled and calculated in FlowJo.

### Bioinformatics analysis

The expression levels of XRCC1 in normal pancreatic tissue and PAAD tissues were analyzed by interrogating 167 samples (GTEx normal), 4 tumor-adjacent tissues (TCGA, 179 carcinoma samples (TCGA). The read counts were normalized to reads per million and then log2 transformed. The R package ggplot2 (version 3.3.3) in the R language is used for visualization. The Mann-Whitney U test (Wilcoxon Rank sum test) was used to compute *p* value for the difference in means.

### Statistical analysis

All measurement data were expressed as the as the mean ± standard error of the mean (SEM), and all statistical analyses were performed using the statistical software GraphPad Prism version 8 (GraphPad Software, Inc.; San Diego, CA, USA). All data satisfied both the normality and equal variance criteria for using parametric tests. The one-way analysis of variance (ANOVA) followed by Tukey’s multiple comparison test was used to test for significance, and a *P* value < 0.05 was considered statistically significant.

## Results

### β-Lap-induced cytotoxicity in pancreatic cancer cells is NQO1-dependent

**Fig. 1:**
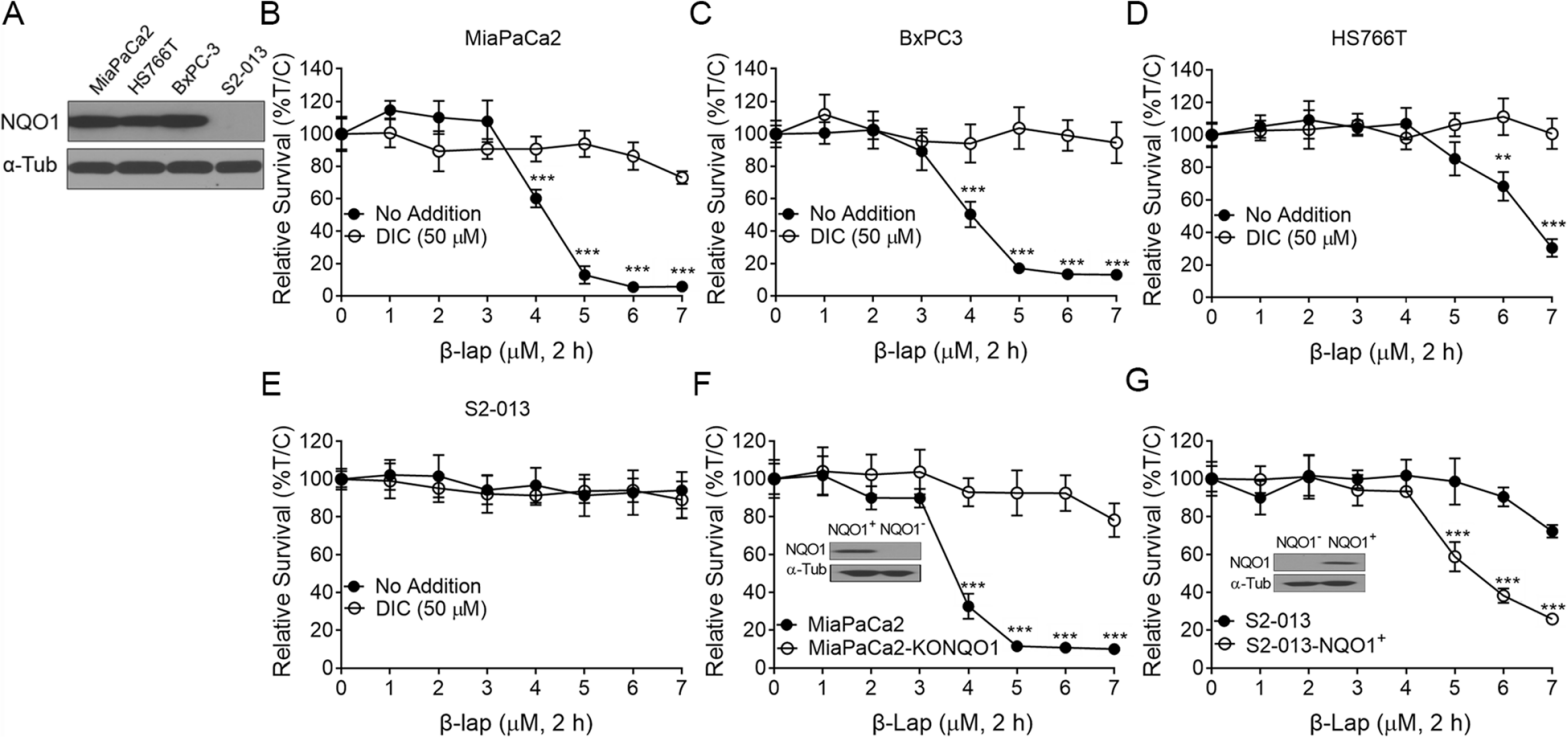
**β-lap induced lethality in NQO1**^**+**^ **PDA Cells**. A. NQO1 expression were measured in Pancreatic cancer cells (MiaPaCa2, BxPC3, HS766T, or S2–013 cells). B-E. PDA cells (MiaPaCa2, BxPC3, HS766T, or S2–013 cells) were treated with or without various β-lap doses (μmol/L, 2 h), ± dicoumarol (50 μmol/L, 2 h). Lethality was monitored by relative survival. Control cells were treated with identical DMSO concentrations (< 0.05%). F. MiaPaCa2 cells knocked out for NQO1 expression (see Western, inset) were treated with β-lap as in (B) and survival were assessed. G. NQO1^+^ S2–013 cells harboring a CMV-NQO1 over-expression vector (see Western, inset) were treated as in (F) and survival were assessed. All error bars are means of six replicates from three independent experiments; means ± SEM. *** *p* < 0.001; ** *p* < 0.01; * *p* < 0.05

To test tumor specificity of β-lap, pancreatic cancer cells (MiaPaCa2, BxPC3, HS766T, or S2–013 cells) were used to examine the roles of NQO1. Figure 1A showed that NQO1 expression was high in MiaPaCa2, BxPC3 and HS766T cell lines, and undetectable in S2–013 cell line.

Cells were treated with various β-lap doses (μmol/L, 2 h), ± dicoumarol (50 μmol/L, 2 h). Lethality was monitored by relative survival. A significant concentration-dependent lethality was observed in MiaPaCa2, BxPC3 and HS766T cells (Figs. 1B~ D), and cells showed lethality at ≥5 μmol/L in MiaPaCa2 and BxPC3 (Fig. 1B and Fig. 1C), and at ≥7 μmol/L in HS766T cells (Fig. 1D). For all four tested pancreatic cancer cell lines, cell death happened in an NQO1-dependent manner, as dicoumarol (DIC, an NQO1 inhibitor) treatment (Figs. 1B~ D) or S2–013 cells (NQO1-deficient cells, Fig. 1E) remained nonresponsive to β-lap, suggesting low NQO1 expression spared cells from lethality induced by β-lap. To further verify β-lap-induced cell death dependent on NQO1 expression, MiaPaCa2-KONQO1 cells and NQO1^+^- S2–013 cells were exposed to β-lap for 2 h. DNA survival assay revealed MiaPaCa2 cells knocked out for NQO1 expression were resistant to β-lap (Fig. 1F), while exogenous over-expression of NQO1 in S2–013 enhanced β-lap lethality (Fig. 1G), consistent with an NQO1-dependent mechanism [7]. Taken together, these results demonstrated that the sensitivity to β-lap was associated with the expression of NQO1.

### XRCC1 loss enhances β-lap-induced DNA damage

**Fig. 2:**
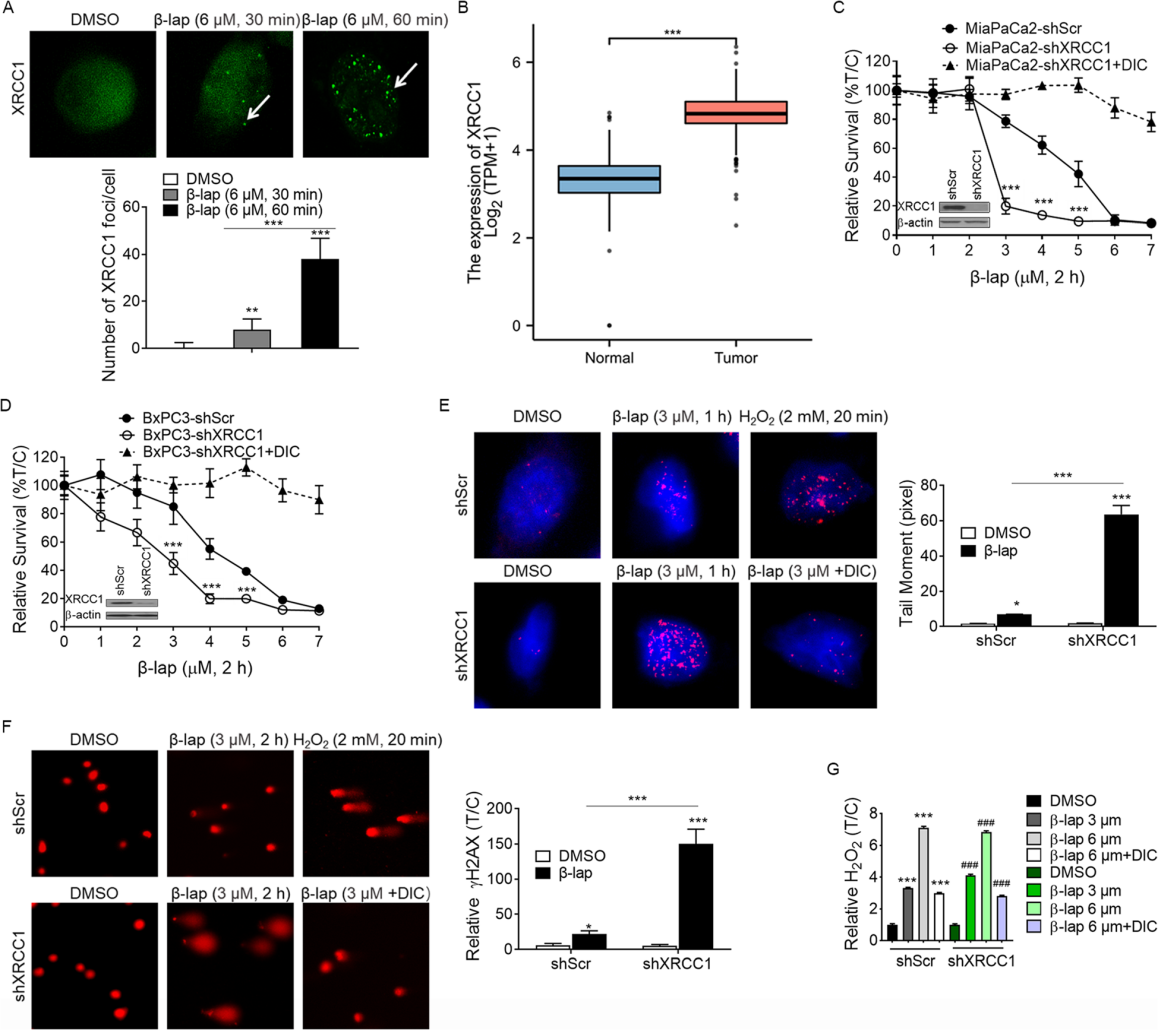
**β-lap exposure results in significant DNA base damage.** A. Distribution patterns of XRCC1 in untreated MiaPaCa2 cells and cells treated with 6 μM of β-lap for 30 min or 60 min. B. Relative mRNA expression levels of *XRCC1* from PAAD tumor (*n* = 179) and associated normal pancreas (*n* = 171) samples. C&D. Relative survival of MiaPaCa2 shXRCC1 and BxPC3 shXRCC1 cells treated with β-lap for 2 h. E. Immunostaining of γH2AX (*red*) in shScr and shXRCC1 MiaPaCa2 cells was monitored after 1 h treatment of 3 μM β-lap with or without dicoumarol (50 μmol/L, 2 h). Nuclei were counterstained with DAPI. bar 10 μm. F. MiaPaCa2 cells were treated with 3 μM β-lap for 1 h with or without dicoumarol (50 μmol/L, 2 h) before performing Comet Assay (alkaline electrophoresis conditions, 23 V for 30 min). Cells were exposed to 2 mM H_2_O_2_ for 20 min (in PBS) as positive control. Comet tail moment were measured by CASP software. Shown are representative images of experiments performed at least three times. Shown are comet tail means ±SEM of at least 50 comet tails per condition. *** *p* ≤ 0.001; ** *p* ≤ 0.01; * *p* ≤ 0.05. G. Relative H_2_O_2_ levels in MiaPaCa2 cells treated with or without various β-lap doses (μmol/L, 2 h), ± dicoumarol (50 μmol/L, 2 h). Values are mean ± S.D. *n* = 3. *** p ≤ 0.001; ** p ≤ 0.01; * p ≤ 0.05 compared with shScr controls. _###_ p ≤ 0.001_; ##_ p ≤ 0.01; _#_p ≤ 0.05 compared with shXRCC1 controls

Previous reports showed that β-lap could generate increased levels of DNA lesions [7]. XRCC1 has been reported to act as a scaffolding protein to mediate BER signaling at the sites of DNA lesions, typically an AP sites or SSBs21 [10]. To test the role of XRCC1 in DNA repair, we treated MiaPaCa2 cell with 6 μmol/L β-lap for 30 min or 60 min. XRCC1 was noted to localize in small and circular foci corresponding to β-lap treatment (Fig. 2A, white arrows showing foci), and 60 min treatment with β-lap generated accumulated and brighter XRCC1-foci, suggesting the recruitment of XRCC1 involved in the repair of β-lap-induced DNA damage. Interestingly, XRCC1-foci became undetectable at 2 h treatment (data not shown). It is unclear if XRCC1 dissociated from the damage site at 2 h treatment is due to accumulated double-strand breaks (DSBs). Moreover, we examined *XRCC1* mRNA expression in normal pancreatic tissue and pancreatic cancer (PAAD) tissues by interrogating 167 samples (GTEx normal), 4 tumor-adjacent tissues (TCGA), 179 carcinoma samples (TCGA) and the results showed that *XRCC1* mRNA expression was significantly elevated in PAAD tissues relative to associated normal pancreatic tissue (Fig. 2B). These data suggest that high levels of *XRCC1* causing BER resistance may be a major problem for β -lap therapy. Since there are currently no known XRCC1 inhibitors, to further explore a possible role of XRCC1 in the sensitivity to β-lap in PDA cells, we knocked down over 90% of the expression of *XRCC1* mRNA in the MiaPaCa2 cells and BxPC3 cells through pLVX-shXRCC1 (Fig. 2C&D) and then evaluated β-lap-induced lethality by relative survival. As shown in Fig. 2C&D, XRCC1 loss significantly increased sensitivity to β-lap in MiaPaCa2 cells and BxPC3 cells, especially in MiaPaCa2 cells. Relative survival assay revealed XRCC1 loss increased lethality induced by β-lap (3 μM, 2 h) from 80% survival in shScr MiaPaCa2 cells to 20% survival in shXRCC1 MiaPaCa2 cells (Fig. 2C). Figure 2C&D also revealed the lethality caused by XRCC1 loss + 3 μmol/L β-lap was largely blocked by DIC treatment.

Given the importance of the recruitment of endogenous XRCC1 at sites of DNA damage to repair DNA damage, we hypothesized that XRCC1 loss resulted in the inhibition of DSB repair, and the sharp response of shXRCC1 MiaPaCa2 cells to β-lap was due to accumulated DNA damage as compared with shScr cells. Therefore, γH2AX foci formation and alkaline comet assay were used to assess the extent of total DNA lesions. shScr and shXRCC1 MiaPaCa2 cell were treated with a sublethal dose of β-lap (3 μM) for 1 h. H_2_O_2_ (2 mM, 20 min) was used as a positive control. Representative images demonstrated that β-lap treatment of shXRCC1 MiaPaCa2 cell resulted in dramatically increased DSB formation as measured by γH2AX foci after 60 min of treatment with β-lap (3 μM), which was blocked by DIC treatment. In contrast, exposure of shScr MiaPaCa2 cell to 3 μM β-lap only caused relative lower levels of γH2AX foci formation at 60 mins (Fig. 2E), which was consistent with our previous finding in survival assay (Fig. 2C) that 3 μM β-lap is a sublethal dose in MiaPaCa2 cells, and a lethal dose in shXRCC1 MiaPaCa2 cells.

Results from alkaline comet assays further supported above findings. As shown in Fig. 2F, the lack of XRCC1 after 3 μM β-lap for 2 h significantly increased the comet tail lengths. Considering β-lap-induced DNA damage was related to ROS production, we then measured β-lap-induced ROS levels in shScr and shXRCC1 MiaPaCa2 cells. MiaPaCa2 cells were exposed to sublethal or lethal β-lap doses for 2 h, and we used H_2_O_2_ as an indicator of ROS to show β-lap-induced ROS. Figure 2G indicated H_2_O_2_ formation in MiaPaCa2 cells was dose-dependent, with 6 μM β -lap (a lethal dose in MiaPaCa2 cells) causing H_2_O_2_ levels that were approximately 2 times to 3 μM β -lap (a sublethal dose in MiaPaCa2 cells), 6.5 times to control group (Fig. 2G), and dicoumarol (DIC) significantly suppressed β-lap-induced H_2_O_2_ formation. Similar results were observed in stable shXRCC1 depletion cells. These combined data revealed that XRCC1 loss might have no effect on β-lap-induced H_2_O_2_ production, but resulted in significant DNA lesions, strongly suggesting that XRCC1 plays a key role in BER to process the DNA damage.

### β-Lap induced S and G2/M phase cell cycle arrest

**Fig. 3:**
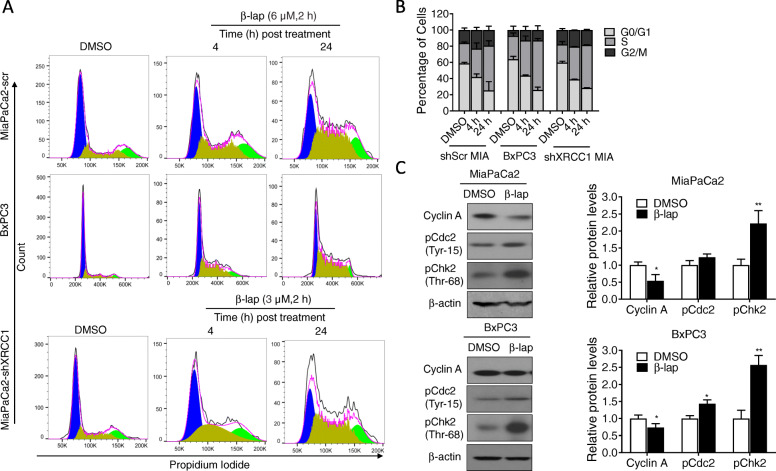
**β-lap blocks MiaPaCa2 and BxPC3 cells in S phase and G2/M phase.** A. MiaPaCa2 cells, BxCP3 and MiaPaCa2-shXRCC1 cells were treated with β–lap (6 μM, 6 μM and 3 μM, respectively). After 2 h treatment, medium containing β-lap was replaced by normal culture medium and then the DNA content was quantitated at 4 or 24 h posttreatment with β-lap by flow cytometry. B. The percentages of cells occupying G1, S, and G2/M phases of the cell cycle are shown. Results are represented as the mean ± SD from three independent trials. C. The effect of β-lap on the expression of cycle-associated proteins in MiaPaCa2 and BxPC3 cells was detected by Western blot after treatment with 6 μM β-lap for 2 h. β-actin was used as a loading control

Because β-lap induced DNA damage, we next examined the effects of β-lap on the cell cycle distribution of PDA cells by performing flow cytometry assays. MiaPaCa2 cells and BxPC3 cells were treated with β-lap. After 2 h treatment, medium containing β-lap was replaced by normal culture medium and then the DNA content was quantitated at 4 or 24 h posttreatment with β-lap by flow cytometry of cells stained with propidium iodide (PI). Results showed that MiaPaCa2 and BxPC3 cells treated with 6 μM β-lap led to accumulation of S phase cells (from 35.12 ± 7.06% at 4 h to 55.28 ± 6.14% at 24 h vs. 25.07 ± 1.37% in the untreated MiaPaCa2 cells; from 43.48 ± 5.45% at 4 h to 61.23 ± 2.43% at 24 h vs. 28.85 ± 3.78% in the untreated BxPC3 cells), which was associated with a significant decrease of cells in the G0/ G1 phase (from 41.87 ± 3.75% at 4 h to 25.36 ± 10.89% at 24 h vs. 58.79 ± 1.25% in the untreated MiaPaCa2 cells; from 43.64 ± 0.94% at 4 h to 25.90 ± 3.66% at 24 h vs. 63.80 ± 3.60% in the untreated BxPC3 cells)(Fig. 3A-B). Moreover, the proportion of cancer cells in G2/M-phase increased slightly (from 22.99 ± 3.30% at 4 h to 19.36 ± 4.75% at 24 h vs. 16.13 ± 2.63% in the untreated MiaPaCa2 cells; from 12.86 ± 4.51% at 4 h to 12.86 ± 5.36% at 24 h vs. 7.34 ± 4.74% in the untreated BxPC3 cells) compared to untreated cells. Similar results were found in shXRCC1 MiaPaCa2 cells treated with 3 μM β -lap. Together, these results suggested that β-lap could cause S as well as G2/M arrest in PDA cells.

Additionally, we investigated the effects of β-lap on cell cycle-related proteins. Western blot analysis showed that β-lap treatment for 2 h elevated the protein levels of phospho-Chk2 (Thr68) and phospho-Cdc2 (Tyr15), along with downregulating the protein expression of Cyclin A (Fig. 3C), which are known as key cell cycle regulators. Taken together, β-lap inhibited PDA cell proliferation by inducing cell cycle arrest at the S and the G2/M checkpoint.

### XRCC1 is a key modulator of β-lap-induced PAR synthesis

**Fig. 4:**
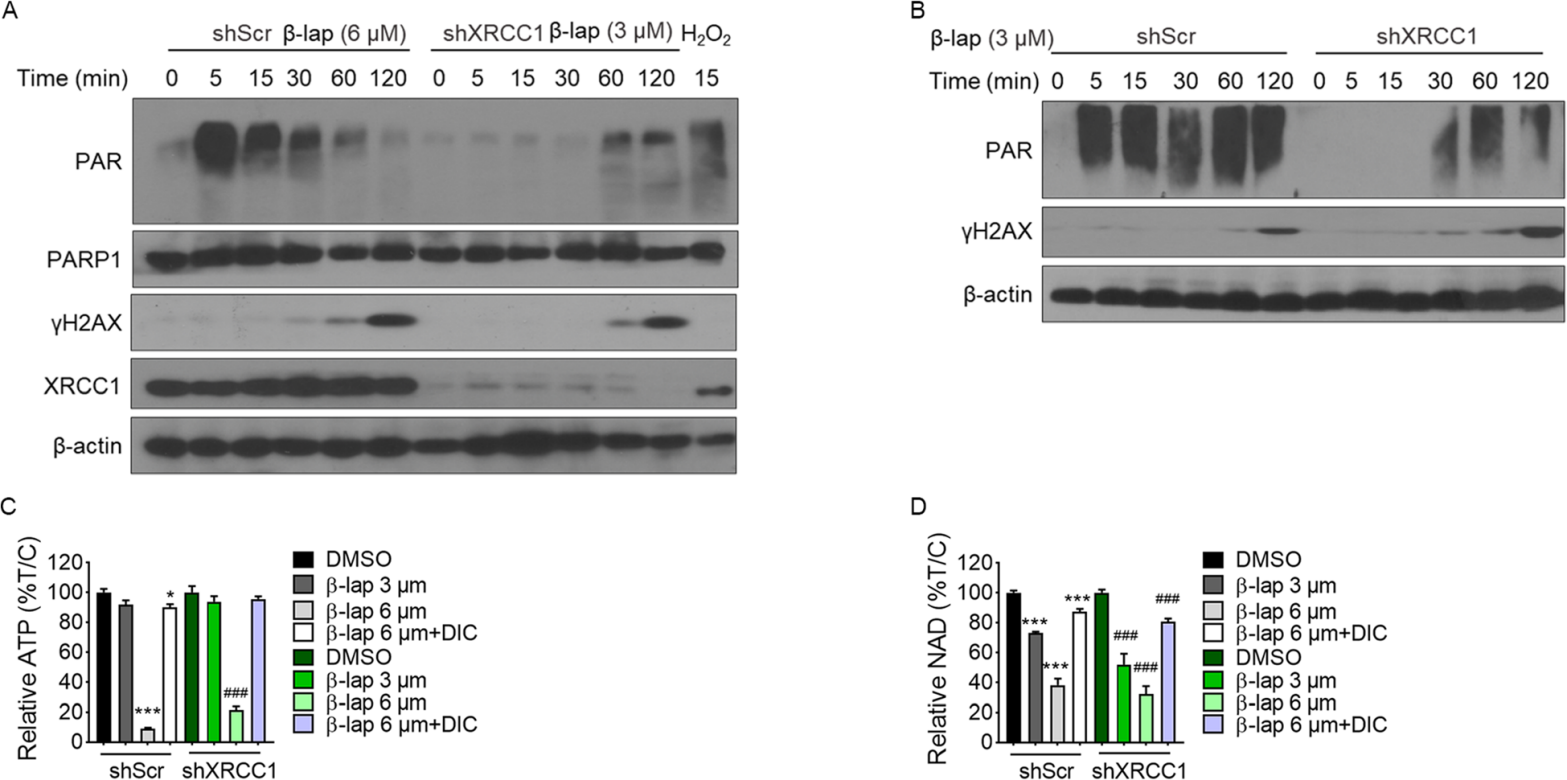
**XRCC1 plays a key role in β-lap-induced PAR synthesis**. A. MiaPaCa2 cells were treated with β-lap (6 μM/ 3 μM) for indicated times. B. MiaPaCa2 cells were treated with β-lap (3 μM) for indicated times. C. Relative ATP levels in MiaPaCa2 cells treated with or without various β-lap doses (μmol/L, 2 h), ± dicoumarol (50 μmol/L, 2 h). D. Relative NAD levels in MiaPaCa2 cells treated with or without various β-lap doses (μmol/L, 2 h), ± dicoumarol (50 μmol/L, 2 h). *** p ≤ 0.001; ** p ≤ 0.01; * p ≤ 0.05 compared with shScr controls. _###_ p ≤ 0.001_; ##_ p ≤ 0.01; _#_ p ≤ 0.05 compared with shXRCC1 controls

One of the earliest events of cellular response to DNA damage is DNA damage-induced auto-PARlyation, a process of poly (ADP-ribose) polymerase 1 (PARP1) catalyzing the synthesis of poly (ADP-ribose) (PAR) polymers at sites of DNA damage. As a scaffold protein, XRCC1 acts to facilitate poly (ADP-ribose) polymerase in DNA repair [12]. Therefore, cellular PAR levels were determined in the stable shXRCC1 depletion MiaPaCa2. Western blotting analysis revealed the PAR level appeared to peak at the 5 min time point and last 60 min in shScr MiaPaCa2 cells treated with 6 μM β-lap (Fig. 4A), consistent to the previously reported that lethal dose of β-lap treatment could cause PARP-1 hyperactivation [8]. In contrast, there was minimal PAR accumulation in XRCC1 depleted cells over the 60 min period after 3 μM β-lap exposure. To further test whether PAR level was regulated by XRCC1, we measured the PAR level in shScr vs shXRCC1 MiaPaCa2 cells exposed to same concentration β-lap (3 μM). As shown in Fig. 4B, there is a lower level of PAR in XRCC1-deficient cells, suggesting XRCC1 is a key modulator of β-lap-induced PARP1 hyperactivation. Additionally, western results show that shXRCC1 cells exposed to β-lap (3 μM) was accompanied by earlier and significantly greater γH2AX level, compared to shScr cells (Fig. 4B), consistent to γH2AX Immunofluorescence results (Fig. 2C), indicating that XRCC1 loss enhanced DNA damage induced by β-lap.

As reported previously PARP1 hyperactivation caused NAD^+^ and ATP dramatic depletion, resulting in cell death [8], total intracellular ATP levels and NAD^+^ were measured after a 2 h treatment with β-lap. Figure 4C-D showed β-lap induced a dose-dependent consumption in NAD^+^ and ATP. MiaPaCa2 cells treated with 6 μM β -lap (a lethal dose in MiaPaca2 cells) displayed a dramatic consumption rate in NAD^+^ and ATP, while exposure to a sublethal dose of β -lap (3 μM) caused no obvious ATP loss and mild decreased NAD^+^. Similarly, NAD^+^ and ATP depletion were also noted in shXRCC1 cells treated with 6 μM β-lap. Of note, NAD^+^ level in 3 μM β-lap-treated shScr cells was higher as compared to 3 μM β-lap-treated shXRCC1 MiaPaCa2 cells (73.13 ± 0.93% in shScr cells vs. 51.98 ± 7.26% in shXRCC1 cells), suggesting XRCC1 loss leading progressively to NAD^+^ depletion induced by β-lap. The ATP/NAD^+^ loss of shScr cells or shXRCC1-depleted cells induced by β-lap was largely abolished by dicoumarol, the NQO1 inhibitor.

### XRCC1 loss provides a molecular switch, converting programmed necrosis to apoptosis

According to previous reports, β-lap induced programmed necrosis caused by dramatic depletion of ATP and NAD^+^. Figure 4C & D showed MiaPaCa2 cell exposed to 3 μM β-lap caused no significant loss of ATP compared to 6 μM β-lap treatment. We subsequently investigated whether β -lap (3 μM) induced necrotic cell death in shXRCC1 cells, as determined by flow cytometry (Annexin-V*/*PI staining) and Western blot analysis.

**Fig. 5:**
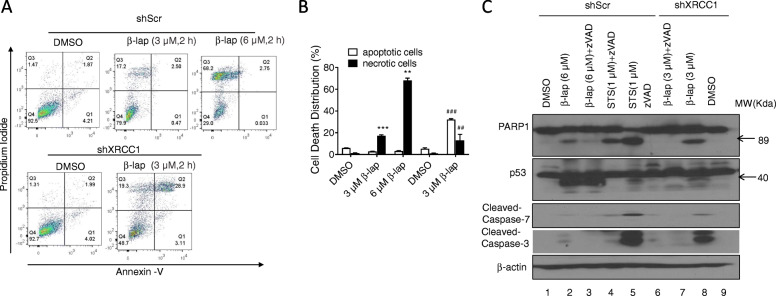
**XRCC1 knock-down induced apoptosis in MiaPaCa2 cells.** A. MiaPaCa2 cells were treated with β-lap (6 μM/ 3 μM) for 2 h and then analysis of cell death in MiaPaCa2 cells (control and *XRCC1* knocked-down) was performed at 24 h posttreatment by flow cytometry. B. Quantitation of the percentage of apoptotic & necrotic cells using the Annexin V assay. Values on the graph represent mean ± SD of three independent experiments. *** p ≤ 0.001; ** p ≤ 0.01; * p ≤ 0.05. (C) Western blot analysis of cleaved apoptotic & necrotic proteins in β-lap–treated *XRCC1* knocked-down and control MiaPaCa2 cells

MiaPaCa2 cells were exposed to β-lap (3 or 6 μM) for 2 h, further incubated in drug-free medium for 24 h, and then analyzed for cell death by Annexin V-FITC/PI double staining and flow-cytometric analysis. Annexin-V^+^*/*PI^−^ (Q1 quadrant) and Annexin-V^+^*/*PI^+^ (Q2 quadrant) were considered to represent early apoptotic cells and late apoptotic cells respectively, and apoptotic cells were counted as both of late and early apoptotic cells. Annexin-V^−^*/*PI^+^ (Q3 quadrant) and Annexin-V^−^*/*PI^−^ (Q4 quadrant) were considered to represent necrotic cells and living cells respectively. After treatment with β-lap, the number of viable shSCR MiaPaCa2 cells was reduced, whereas the numbers of necrotic cells were significantly increased (17.2 ± 0.90% in 3 μM β-lap-treated group; 68.01 ± 2.19% in 6 μM β-lap-treated group) in a significant dose-dependent manner (Fig. 5A–B). In contrast to β-lap-induced necrotic cell death of shScr MiaPaCa2 cells, a dramatic increase was detected in apoptotic cells (32.11% ± 1.21%, *P* < 0.001) in β-lap-treated shXRCC1 MiaPaCa2 cell when compared with the shScr MiaPaCa2 cells treated with the same dose of β-lap (3 μM) (2.85 ± 0.25%) (Fig. 5A–B), suggesting XRCC1 loss resulted in significant enhancement of apoptosis of MiaPaCa2 cells.

Western blot assay showed that treatment of shScr MiaPaCa2 cells with a lethal dose of β-lap alone (6 μM) resulted in p53 proteolytic cleavage, diagnostic of programmed necrosis (lane 2, Fig. 5C), while cleavage of caspase 3/7 and cleaved PARP1 (89 kDa), diagnostic of apoptosis, were found in shXRCC1 cells exposed to 3 μM β-lap (lane 8, Fig. 5C), consistent to flow cytometry analysis. Active caspase7 and active caspase3 play critical roles in the regulation of apoptosis which cleave a broad spectrum of cellular target proteins, including PARP1, thus leading to cell death [13]. To further address the significance of caspase activation in β-lap-induced apoptosis, we examined the effects of z-VAD-fmk (a pan-caspase inhibitor) and used Staurosporine (STS)-induced apoptosis as positive control. Figure 5C (lane 5) showed STS yielded significantly classic apoptosis-related ~ 89 kDa PARP1 proteolysis and Caspase 3/7 activation, similarly to the responses of shXRCC1 cells to β-lap. The pan-caspase inhibitor, z-VAD-fmk, attenuated the expression of cleaved PARP1/ Caspase 3/7 induced by STS (lane 4, Fig. 5C). β-lap alone (6 μM)-treated shScr MiaPaCa2 cell preincubated with z-VAD-fmk, had no influence on p53 proteolytic cleavage but block β-lap-induced weak PARP1 proteolysis (lane 3, Fig. 5C). In contrast, cleavage of caspase 3/7 /cleaved PARP1 (89 kDa) found in shXRCC1 cells were efficiently blocked by zVAD-fmk (lanes 7 vs 8, Fig. 5C). These results indicated that *XRCC1* knockdown in MiaPaCa2 cells might trigger a transition of cell death mode to apoptotic cell death.

## Discussion

NQO1 is overexpressed in most solid cancers and lacking in control healthy cells. Increased NQO1/decreased catalase expression is observed in pancreatic intraepithelial neoplasia, precursor lesions of pancreatic cancer [5, 14]. Thus, NQO1 bioactivatable drugs have been ideally suited for anticancer therapy. Here we focus our studies on the specific NQO1 bioactivatable drug, β-lap (ARQ761 in clinical form). β-lap treatment has been received great promise in NQO1 overexpressed tumors. Although promising, prior clinical trial data suggested that it is necessary to reduce dose-limiting toxicity [15, 16]. In our pretrial β-lap of maximum tolerate dose was 30 mg/kg for IV injection in mice. After dosing of about 30 mg/kg, mouse immediately got labored breathing and an irregular gait. If using more than 30 mg/kg caused severe muscle contractions, labored breathing, and lethality in some cases (data not shown). In the current study, we knockdown *XRCC1*, combined with β-lap to treat PDA cells in vitro.

XRCC1, a 70- kDa protein, rapidly responds to DNA damage in cells [10], which may serve as a strand-break sensor [17, 18]. Once XRCC1 is bound to nicked and gapped DNA, it serves as a scaffold protein able to coordinate and facilitate the downstream SSBR enzymes. A number of studies showed *XRCC1*^*−/−*^ cells were significantly more sensitive to alkylating agents and IR [9, 19]. Here, exposure of MiaPaCa2 cell to β-lap induced XRCC1 foci formation, strongly suggesting XRCC1 involved in DNA repair caused by β-lap. *XRCC1*-knockdown MiaPaCa2 cells exposed to β-lap led to decreased survival rates, consistent with enhanced effects on γH2AX foci formation and tail moments of Comet assays. These data indicated XRCC1 loss amplifies DNA damage induced by β-lap, consistent with Chakrabarti et al. report that XRCC1 depletion strongly increased hypersensitivity to β-lap [14]. To understand the potential cellular events involving the antiproliferative activity of β-lap, PDA cells exposed to β-lap and cell cycle analysis was performed. β-lap treatment caused S as well as G2/M arrest in PDA cells, consistent with previous reports indicating that β-lap induced a cell cycle arrest in S phase/ G2/M-phase in diverse cancer cell lines [20–22], and *XRCC1* knockdown had no detectable effect on cycle.

Inconsistent data have been published on β-lap effects on NQO^+^-cell death. β-lap has been reported to induce multiple cell death mechanisms including autophagy, apoptosis and necrotic cell death in NQO1^+^-cancer cells [23–26]. Our data indicated that DNA damage created by high dose of β-lap caused PARP1 hyperactivation, leading to the massive synthesis of poly (ADP-ribose) (PAR) from nicotinamide adenine (NAD^+^) and subsequent rapid NAD ^+^/ATP depletion, and, in consequence resulting in programmed necrosis, which was similar to some reports [7, 15].

The interaction of XRCC1 with poly (ADP-ribose) (PAR) is essential for XRCC1 functionality during BER [27]. Several reports have suggested that PAR synthesis at DNA single strand breaks can promote the recruitment of XRCC1 [12, 27, 28]. Howerer, conflicting data have been published on the absence of XRCC1 protein resulting in stimulatory or inhibitory effects on PAR formation [12, 29]. The inconsistence is likely due to different DNA damaging agents and methods used in their studies. Our work confirms the latter report [29] showing that XRCC1 loss results in excessive NAD^+^ depletion (Fig. 4D), then declining PAR synthesis. These data suggest that XRCC1 recruitment is not only a downstream effector, but also an upstream regulator of PAR formation. PAR synthesis has been reported to induce caspase-independent cell death such as necroptosis or parthanatos [30, 31]. Moreover, our data showed XRCC1 loss significantly lowered the efficacious dose of β-lap. Additionally, lower concentration of β-lap treatment combined with *XRCC1* knockdown caused mild NAD+ & ATP loss compared to higher concentration of β-lap treatment. Considering the difference observed in PAR synthesis and NAD^+^ & ATP consumption, we next determined the effects of XRCC1 loss on cell death by β-lap. Perhaps due to high ATP levels typically enable a cell to undergo apoptosis, whereas low ATP levels favor necrosis. Therefore, compared to high dose (6 μM) of β-lap treatment, higher ATP levels and decreased PAR synthesis in 3 μM β-lap-treated XRCC1-deficient MiaPaCa2 cells switched necrosis to the energy requiring & caspase-dependent apoptosis.

In summary, our data showed combining XRCC1 loss with β-lap led to NQO1-dependent SSBs, DSBs and apoptosis & necrosis in vitro. Although more in vivo studies are needed to elucidate therapeutic effects and the exact mechanism, our data identified targeting the enzymes in DNA repair pathways for therapeutic treatment may, have the potential to synergize with β-lap for treating human NQO1+ cancer.

## Conclusions

Our study supports the existing perception that β-lap is an NQO1 bioactivatable drug, and β-lap’s lethality stems from β-lap-induced futile redox cycling in NQO1+ cancer cells, producing oxidative DNA lesions and consequent PARP1- mediated necrotic cell death. We found that XRCC1 was involved in the repair of β-lap-induced DNA damage, and XRCC1 loss amplified DNA damage induced by β-lap. Moreover, our date showed that β-lap treatment with XRCC1 loss lowered the efficacious dose of β-lap and switched necrosis to caspase-dependent apoptosis. We also studied the association between XRCC1 and PAR synthesis, and we found XRCC1 loss reduced PAR synthesis, which demands further studies.

## Supplementary Information

**Additional file 1 Supplementary Figure S6:** Full-length images of blots and gels presented in the main figures.

## Data Availability

The data used or analyzed during this study are available on reasonable request from the corresponding author (lycmellisa@126.com).

## Abbreviations

BER: Base excision repair
DIC: Dicoumarol
DSBs: Double strand breaks
NAD: Nicotinamide adenine
NQO1: NAD(P) H quinone oxidoreductase 1
PARP1: PolY (ADP-RIBOSE) POLYMERASE 1
PDA: PANCREATIC DUCTAL ADENOCARCINOMA
ROS: REACTIVE OXYGEN SPECIES
SSBS: Single strand breaks
STS: Staurosporine
XRCC1: X-ray repair cross-complementing protein 1
β-lap: β-lapachone
