# Supplementary figures and images for: X-ray repair cross-complementing protein 1 (XRCC1) loss promotes β-lapachone –induced apoptosis in pancreatic cancer cells
Source: BMC Cancer. 2021 Nov 17;21:1234. doi: 10.1186/s12885-021-08979-y (PMC8600733; doi:10.1186/s12885-021-08979-y)

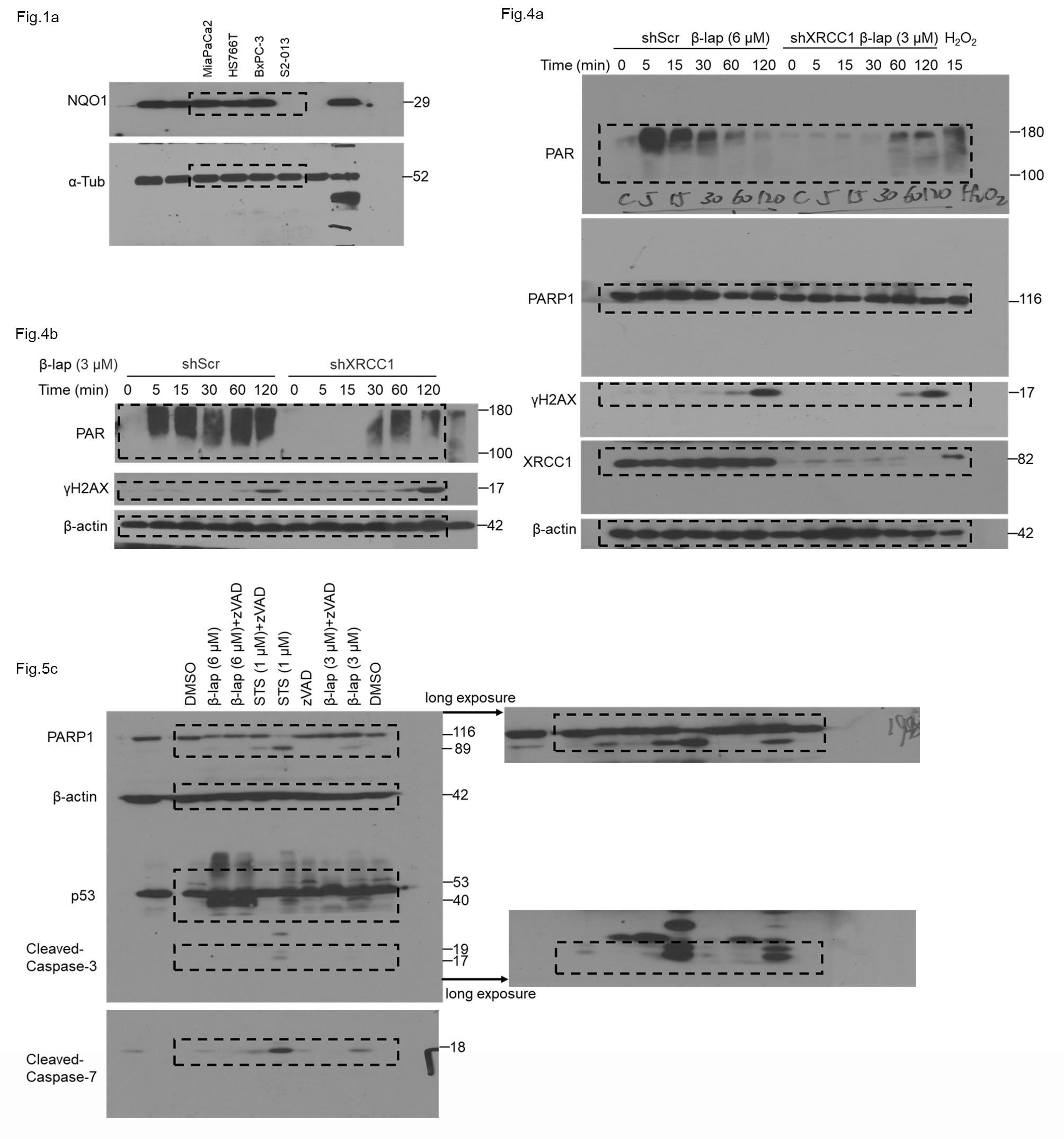

Supplement: Supplementary file 1 — Additional file 1 Supplementary Figure S6: Full-length images of blots and gels presented in the main figures. [file 12885_2021_8979_MOESM1_ESM.tif]
